# Merkel Cell Carcinoma Mimicking a Pyogenic Granuloma on the Thumb

**DOI:** 10.7759/cureus.50878

**Published:** 2023-12-21

**Authors:** Rafik Fanous

**Affiliations:** 1 Family Medicine, Stourport Medical Centre, Stourport-on-Severn, GBR

**Keywords:** silver nitrate cauterization, mcc, granuloma, pyogenic, skin, cancer, merkle cell carcinoma

## Abstract

Merkel cell carcinoma (MCC) is a rare and aggressive skin cancer that can present with various clinical manifestations. An 80-year-old male, known to have chronic lymphocytic leukaemia presented with a lesion on his left thumb, initially thought to be a pyogenic granuloma. The lesion was cauterized with silver nitrate but remained persistent. A curettage biopsy was performed, and a histopathological examination revealed MCC. The patient was subsequently referred to dermatology and plastic surgery for further management. The lesion was excised; a full-thickness skin graft was used to close the defect. This case highlights the importance of considering MCC in the differential diagnosis of skin lesions, even if they present with a benign appearance.

## Introduction

Merkel cell carcinoma (MCC) is a rare, aggressive cancer which is derived from Merkel cells. These are specialized neuroendocrine cells located in the basal layer of the epidermis [1]. MCC most commonly affects elderly individuals and is associated with a high mortality rate. The clinical presentation of MCC can vary, but it typically presents as a fast-growing skin or subcutaneous swelling, usually located on a sun-exposed skin area [2]. The main risk factors are old age, sun exposure, ultraviolet rays, immunosuppressed patients, lymphoproliferative malignancies, and transplant patients. Also, an increased risk of MCC is found in Caucasians [3]. The diagnosis of MCC is based on histopathological examination, which reveals characteristic features, including small blue cells with scanty cytoplasm and neuroendocrine differentiation [4]. 

## Case presentation

An 80-year-old male presented to the clinic with a lesion on his left thumb that had been present for several weeks. He was a known chronic lymphocytic leukaemia (CLL) patient under active surveillance after being treated with fludarabine and cyclophosphamide to which he showed a very good response. His CT scan showed supra and infra diaphragmatic lymphadenopathy and splenomegaly, which is in keeping with CLL rather than MCC as it did not change from the last CT he had four months ago. The case did not warrant restarting chemotherapy for the CLL. It was decided after a multidisciplinary team (MDT) meeting that there was no need for any radiotherapy.

**Figure 1:**
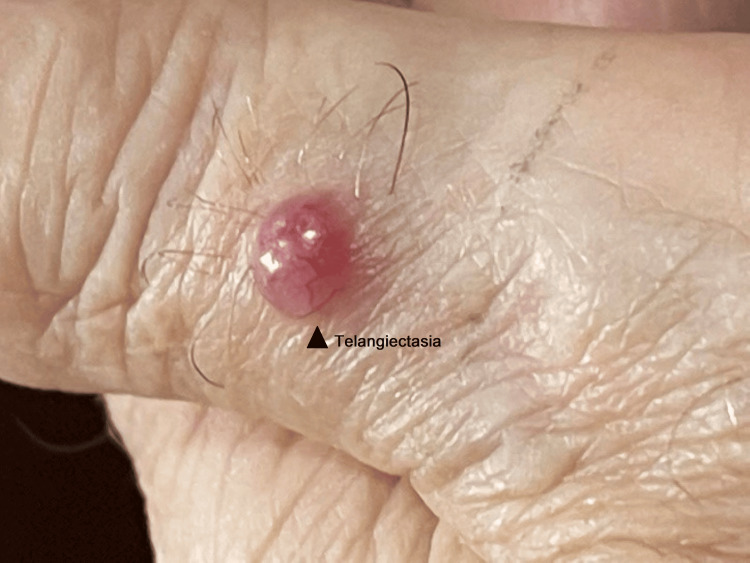
Left thumb with a 1 cm erythematous nodule showing telangiectasia

On examination, a 1 cm erythematous nodule was observed on the dorsal aspect of the patient's left thumb. The nodule had a soft consistency and was non-tender to palpation. No lymphadenopathy in the axillary or the trochlear region was noted. The remainder of the physical examination was unremarkable. The case here presented clinically as a painless, reddish, skin lesion that grew rapidly. The overlying skin did not show any ulceration, but it showed telangiectasia (Figure 1) and acneiform features [1]. Initially, the lesion was thought to be a pyogenic granuloma, a common benign vascular growth, and it was cauterized with silver nitrate sticks as a treatment attempt.

However, despite the intervention, the lesion persisted, prompting further management. The lesion was shaved with a loop curette and sent to histopathology to determine the nature of the lesion. The base of the lesion was cauterized with a hyfrecator to manage bleeding.

**Figure 2:**
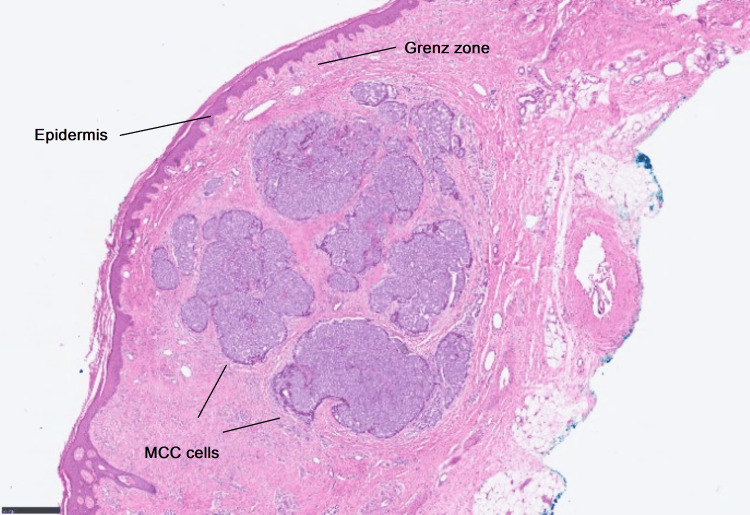
Histopathological picture of the lesion Patient histology slide showing a dermal mass that approaching the subcutis. The epidermis is clear (Grenz Zone).

Histopathological examination of the biopsy specimen revealed MCC, a rare and aggressive form of neuroendocrine skin cancer. Routine studies with both hematoxylin and eosin as well as immunohistochemical stains were done to distinguish MCC from other poorly differentiated tumors. The slide showed a dermal mass that approached the subcutis. The slide showed a dermal mass that approached the subcutis. The epidermis was not involved (Grenz Zone) (Figure 2) [4].

**Figure 3:**
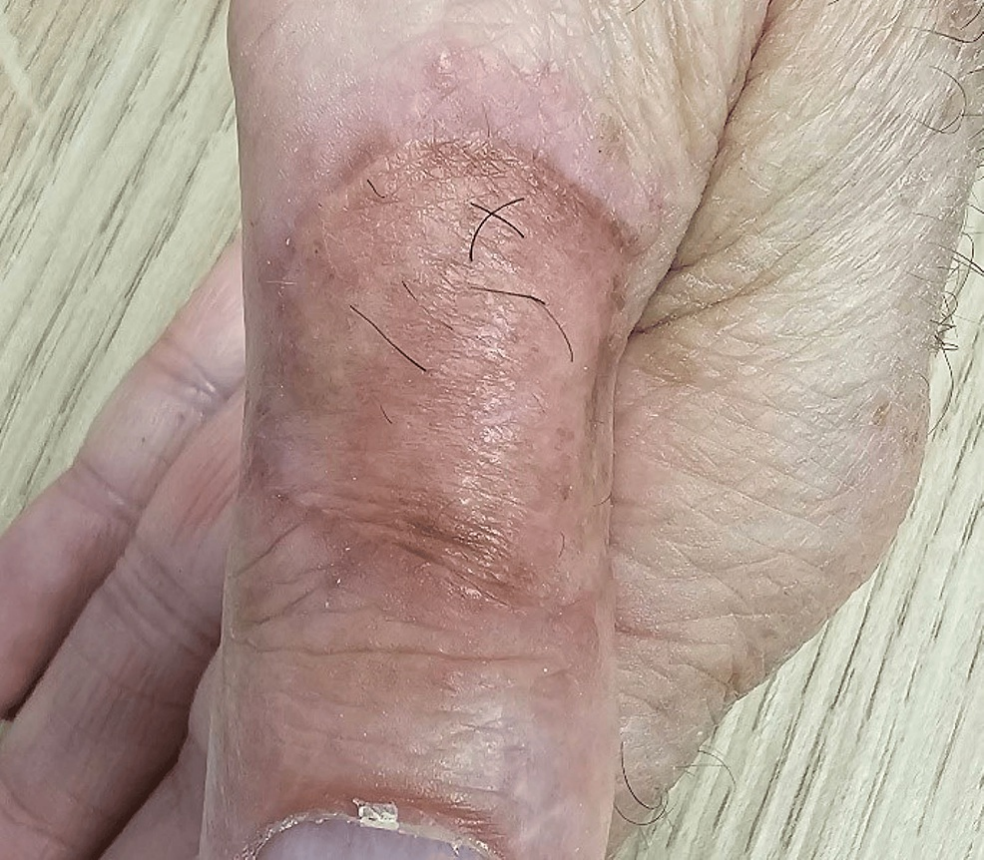
Full-thickness skin graft taken from lower abdomen region showing good healing and coverage.

Following the diagnosis, the patient was promptly referred to dermatology and plastic surgery departments for further management. The lesion was excised with a 1 cm safety margin, and the defect was covered with a full-thickness skin graft (Figure 3) taken from the lower abdomen region. The excised specimen was sent for histopathological examination, which confirmed the diagnosis of MCC with clear margins. This is in keeping with recommendations from the National Comprehensive Cancer Network (NCCN) that states that wide local excision is the gold standard treatment for MCC. It recommends 1-2 cm safety margins all around the lesion circumferentially both at the periphery and deep edge [5].

The patient had an uneventful postoperative recovery and was followed up in the clinic under plastic surgery. No recurrence detected up to six months. He has had six monthly follow-ups and open access to the plastic surgery clinic in case of any changes, new swellings, or concerns. 

## Discussion

MCC is an infrequent and often misdiagnosed skin cancer due to its varied clinical presentations. It can be mistakenly identified as a benign skin lesion like a pyogenic granuloma. This case emphasizes the significance of considering MCC in the differential diagnosis of skin lesions, particularly in elderly individuals or those with a history of extensive sun exposure. In a study by Health et al., they found that the face is the most commonly affected site, followed by the lower limbs and then the upper limbs (Table 1) [6].

**Table 1:** Presentation according to anatomic site Table source: Health et al., 2008 [6]; Used with permission by the author

	Number	Percentage
Primary Skin Lesion	168	85%
Head and neck	56	29%
Lower Limb	46	24%
Upper Limb	40	21%
Trunck	16	8%
Buttock	9	5%
Vulva	1	0.5%
No known Primary (nodal presentation)	27	14%

Several causative factors contribute to the development of MCC, including exposure to ultraviolet (UV) radiation, infection with Merkel cell polyomavirus (MCPyV), chronic immunosuppression, and lymphoproliferative malignancies [3], as in the current case. MCPyV, a human polyomavirus, is linked to MCC in a significant proportion of cases, accounting for approximately 80% of them [7]. Additionally, the case underscores that the majority of MCC diagnoses occur in individuals above the age of 50, primarily in the Caucasian population [1,3].

In Heath et al.'s study of 195 cases between 1980-2007, MCC's most significant features were AEIOU (Asymptomatic, Expanding, Immune suppression, Old (>50 years), Ultra violet-exposed area on fair-skinned individuals). Among the immunosuppressed group, CLL patients represented the highest percentage. The most common provisional diagnosis was a "cyst". This was followed by non-melanoma skin cancer. Other lesions like pyogenic granuloma were presented in 7% of the cases. MCC had the lowest percentage (1%) of provisional diagnosis, which reflects the difficulty of diagnosing the condition [6].

Histopathological examination remains the cornerstone for diagnosing MCC, revealing characteristic features of small blue cells with scant cytoplasm and neuroendocrine differentiation. This emphasizes the importance of histopathological evaluation in confirming the diagnosis and initiating appropriate management strategies.

Wide local excision is the gold standard treatment for MCC with 1-2 cm safety margins all around the lesion circumferentially both at the periphery and deep edge [5]. Radiotherapy has a good role as an adjuvant to surgical treatment, especially if the lesion is more than 3 cm. Despite MCC being considered chemosensitive, chemotherapy offers only short-term response, plus the fact that most of the patients belong to older age groups, chemotherapy is not very well tolerated. On the other hand, immunotherapy is an evolving treatment modality, especially for advanced cases with metastasis, which massively changed care for these cases [3].

## Conclusions

MCC is an uncommon tumour that mainly affects the elderly. The main risk factors are sun and UV exposure, polyomavirus, and immunosuppression. MCC's Initial presentation is very vague and non-specific, which often can be mistaken for a variety of benign and malignant cases as seen in this case, which was initially treated as a pyogenic granuloma. It is a true challenge that can be confusing to the clinician.

There is a limited number of prospective clinical trials conducted to establish treatment guidelines. Consequently, the current recommendations for its treatment primarily rely on retrospective studies involving a small patient population. MCC typically involves surgical excision with a safety margin plus regional lymph node evaluation. Adjuvant radiotherapy may be considered in patients with high-risk features such as large tumours, positive margins, or lymph node involvement. Chemotherapy may be used in the setting of metastatic disease. Immunotherapy is a potential game changer, especially for advanced metastatic cases. The prognosis of MCC is generally poor, with a high risk of local recurrence and distant metastasis.

While surgical excision is the mainstay of treatment for localized MCC, there is ongoing debate over the optimal surgical margin and the role of adjuvant therapy. It would be worthwhile to discuss the different surgical approaches and the evidence behind them, as well as the potential benefits and risks of adjuvant radiotherapy and chemotherapy.
